# Supplementary material for: Role of Compensatory miRNA Networks in Cognitive Recovery from Heart Failure
Source: Noncoding RNA. 2025 Jun 12;11(3):45. doi: 10.3390/ncrna11030045 (PMC12196295; doi:10.3390/ncrna11030045)
Supplement: Supplementary file 1 [file ncrna-11-00045-s001.zip › Supplemental Figures docx.pdf]

Supplemental Figure S1 (Fig S1)

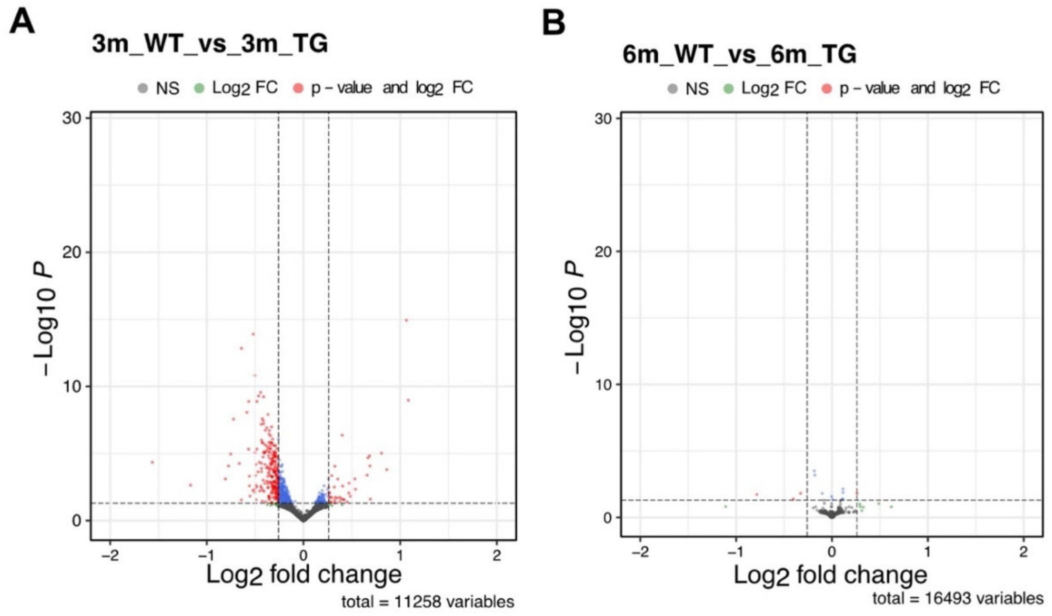

Figure S1. Changes in gene expression in 3 and 6 months-old mice between wild type control and CamKIIδC TG mice. A. Volcano Plots showing differentially expressed genes when comparing WT and TG in 3 months-old mice (n=5, n=6, respectively). **B.** Volcano Plots showing differentially expressed genes when comparing WT and TG in 6 months-old mice (n=11, n=15, respectively).

Supplemental Figure S2 (Fig S2)

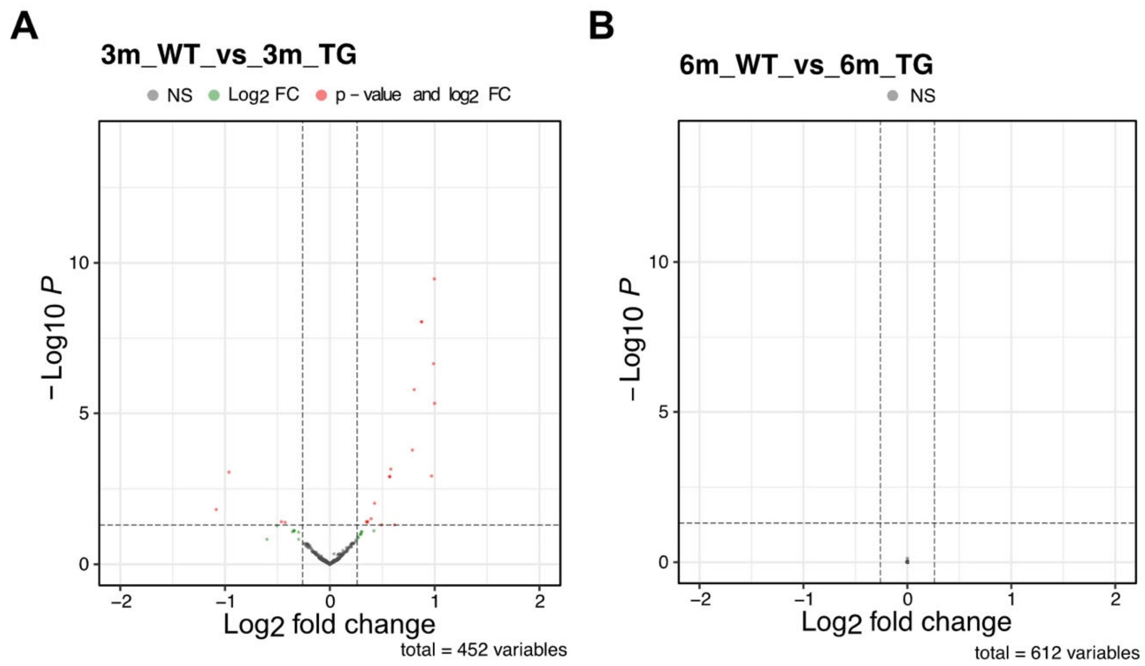

Figure S2. Changes in miRNA expression in 3 and 6 months-old mice between wild type control and CamKIIδC TG mice. A. Volcano Plots showing differentially expressed miRNAs when comparing WT and TG in 3 months-old mice (n=5, n=6, respectively). **B.** Volcano Plots showing differentially expressed miRNAs when comparing WT and TG in 6 months-old mice (n=15, n=16, respectively).
